# Development and validation of a predictive nomogram for cage migration after posterior lumbar interbody fusion: a retrospective study of 517 patients

**DOI:** 10.3389/fsurg.2026.1785695

**Published:** 2026-05-15

**Authors:** Yuan Ma, Jianji Chen, Hongzhen Li, Yuqiao Li, Yuejiao Zhang, Lixin Tang

**Affiliations:** 1 Department of Spine Surgery, Nanyang Central Hospital, Nanyang, Henan Province, China; 2 Department of Spine Surgery, Peking University People’s Hospital, Beijing, China; 3 Department of Spine Surgery, Beijing Tiantan Hospital, Capital Medical University, Beijing, China; 4 Department of Radiology, Shengli Oilfield Central Hospital, Dongying, Shandong Province, China

**Keywords:** cage migration, nomogram, osteoporosis, PI-LL mismatch, posterior lumbar interbody fusion, predictive model, preoperative hemoglobin

## Abstract

**Objective:**

Posterior lumbar interbody fusion (PLIF) is widely used for lumbar diseases, but cage migration (CM) remains a frequent and serious complication. Identifying risk factors for CM is crucial to improving surgical outcomes.

**Methods:**

A retrospective analysis was conducted on 517 patients who underwent PLIF surgery at Nanyang Central Hospital between January 2020 and December 2024. Patients were divided into CM (*n* = 46) and non-CM groups (*n* = 471) based on postoperative imaging findings. Demographic characteristics, intraoperative parameters, and radiographic data were collected. Univariate and multivariate logistic regression analyses were performed to identify independent predictors of CM. A nomogram model was constructed based on significant variables, and its performance was evaluated using a receiver operating characteristic (ROC) curve, calibration curve, and decision curve analysis (DCA).

**Results:**

The overall incidence of CM was 8.9%. Univariate analysis revealed significant differences in the rates of osteoporosis, spondylolisthesis, Modic changes, PI-LL mismatch, and preoperative hemoglobin levels between the two groups (*p* < 0.05). Multivariate analysis identified osteoporosis (OR = 4.186, 95% CI: 1.654–11.421), spondylolisthesis (OR = 8.199, 95% CI: 3.191–22.937), Modic type I change (OR = 8.279, 95% CI: 3.135–23.891), PI-LL (OR = 1.306, 95% CI: 1.192–1.453), and preoperative hemoglobin level (OR = 0.822, 95% CI: 0.753–0.888) as independent risk factors for CM. A nomogram incorporating these five variables showed good discrimination (AUC=0.874, 95% CI: 0.813–0.935) and calibration (Hosmer–Lemeshow test, *χ*^2^ = 7.530, *P* = 0.481). DCA demonstrated a favorable net benefit across a threshold probability range of 0.05 to 0.81.

**Conclusion:**

The developed nomogram model may assist clinicians in preoperative risk stratification and in formulating personalized surgical and perioperative strategies.

## Introduction

Since Cloward et al. first reported posterior lumbar interbody fusion (PLIF) in 1953, PLIF has been widely used in the surgical treatment of lumbar diseases in the history of spinal surgery, such as trauma, deformity, primary and secondary tumors, and infectious lesions (1, 2). In the past few decades, the total number of spinal fusion surgeries has been on the rise worldwide, and PLIF has been widely used in clinical treatment as a classic procedure in lumbar fusion worldwide. According to statistics, the number of primary lumbar fusion surgeries performed each year increased by about 2.7 times from 1998 to 2008 (3). At the same time, the complication rate is 10%–24%, which is much higher than that of ordinary orthopedic surgery (4, 5). Therefore, reducing complications is crucial to improving the prognosis of patients undergoing spinal fusion.

During PLIF surgery, the intervertebral fusion cage (Cage) is used to restore intervertebral height, reconstruct spinal stability, and promote bone fusion. Implanting it into the intervertebral space can restore normal intervertebral height, prevent spinal deformity, and ensure the success of intervertebral fusion (6). However, with the observation of imaging during the perioperative and postoperative follow-up, Cage-related complications, including subsidence, displacement, nerve injury, and pseudoarthrosis, occur from time to time (7). Among them, cage migration (CM) is one of the most common complications. CM may lead to fusion failure, nerve compression, or the need for reoperation. CM may cause progressive spinal deformity and changes in intervertebral disc height. When the Cage moves back into the spinal canal or intervertebral foramen, it will cause direct compression of nerve tissue and neurological dysfunction. In severe cases, revision surgery is required (8). Although previous studies have reported on the impact of CM, the impact of multiple factors on it has not been studied simultaneously (9, 10). Therefore, determining the relevant risk factors to prevent these complications has become a hot issue that needs to be urgently addressed in spinal surgery.

## Patients and methods

### Patient characteristics

The subjects were selected according to the eligibility criteria. The main inclusion criteria: (1) Lumbar PLIF surgery due to lumbar degenerative diseases such as lumbar disc herniation, lumbar spinal stenosis, lumbar spondylolisthesis; (2) The surgical segment is single-segment or double-segment; (3) Complete imaging data before and after the operation. The main exclusion criteria: (1) Previous history of spine, pelvic, or lower limb surgery; (2) Simultaneous history of ankylosing spondylitis, lumbar tuberculosis, lumbar tumor, and spinal fracture; (3) History of lumbar trauma after lumbar surgery; (4) Incomplete imaging data.

### Patient population

This retrospective study analyzed the clinical medical records of patients who underwent lumbar PLIF surgery in Nanyang Central Hospital from January 2020 to December 2024. According to the inclusion and exclusion criteria, a total of 517 patients were included in the study. There were 255 male patients and 262 female patients, with an age range of 43 to 73 years. According to whether CM occurred or not, the enrolled patients were divided into CM and non-CM groups. Because this was a retrospective study based on consecutive eligible patients during the study period, no *a priori* sample size calculation was performed. This study was conducted in accordance with the Declaration of Helsinki and was approved by the Institutional Ethics Review Board of Nanyang Central Hospital (IRB No. 20250308012).

### Surgical techniques

A standard posterior midline approach was used. After a midline skin incision, the paraspinal muscles, including the longissimus and multifidus, were dissected subperiosteally to expose the posterior elements. The posterior bony structures of the operative level, including the laminae, facet joints, and transverse processes, were adequately exposed. At L5-S1, exposure was extended to the sacral ala when necessary. Pedicle screw entry points were identified using the Weinstein technique, and the pedicle screw channels were prepared in the standard fashion. After fluoroscopic confirmation of the trajectory, pedicle screws of appropriate size were inserted.

Laminectomy and decompression were then performed, and the hypertrophic ligamentum flavum was removed to expose the dura and nerve roots. Additional decompression of the lateral recesses and neural foramina was performed along the course of the affected nerve roots when indicated.

After protection of the neural elements, the disc space was entered, and disc material was removed using pituitary forceps, curettes, and reamers. The cartilaginous endplate was carefully removed while preserving the bony endplate to avoid endplate violation. The cage was packed with morselized autologous local bone and allograft and inserted into the intervertebral space under fluoroscopic guidance using a cage inserter. It was then gently advanced to the planned position within the disc space, and its final location was confirmed on anteroposterior and lateral fluoroscopy. Additional morselized bone graft was placed within the disc space around the cage to increase the fusion bed. Pre-bent rods were then applied, and distraction or compression was performed as needed before final tightening of the pedicle screw system. After irrigation and meticulous hemostasis, a drainage tube was placed, and the incision was closed in layers. Postoperative drainage management, neurological monitoring, and brace-assisted mobilization were performed according to the institutional protocol.

### Clinical assessments

CM is generally defined as displacement of the interbody cage greater than 3 mm or beyond the vertebral wall (11). Two spine surgeons independently measured the radiographic parameters, and each measurement was repeated after an interval of at least 1 week. General demographic data, including sex, age, BMI, laboratory parameters, intraoperative variables, and radiographic data, were recorded and analyzed. The mean intraobserver ICC was 0.943, and the mean interobserver ICC was 0.823, indicating good measurement reliability.

### Statistical methods

Statistical analysis was performed using R software(version 4.4.1) and SPSS software(version 22.0). Categorical data were presented as frequencies, while continuous data were expressed as mean ± standard deviation. In univariate analysis, categorical variables were assessed using the chi-square test or Fisher's exact test, while continuous variables were compared between groups using an independent samples t-test or a rank-sum test, depending on the distribution normality. Intraobserver repeatability and interobserver reliability of radiographic measurements were assessed using intraclass correlation coefficients (ICCs). Multivariate logistic regression was performed to analyze factors influencing CM, and OR and 95% CI were calculated. Because PI, LL, and PI-LL are mathematically and clinically interrelated, they were not entered simultaneously into the multivariable model; PI-LL was retained as the representative sagittal alignment variable. A nomogram for evaluating CM risk was constructed using the influencing factors filtered out by multivariate logistic regression. Calibration and DCA curves were constructed to assess the discrimination, calibration, and practicability of the nomogram. A ROC curve was constructed, and bootstrap resampling was repeated 2,000 times to internally verify the nomogram. Differences with *p* < 0.05 were considered statistically significant.

## Results

### Clinical results

**Figure 1:**
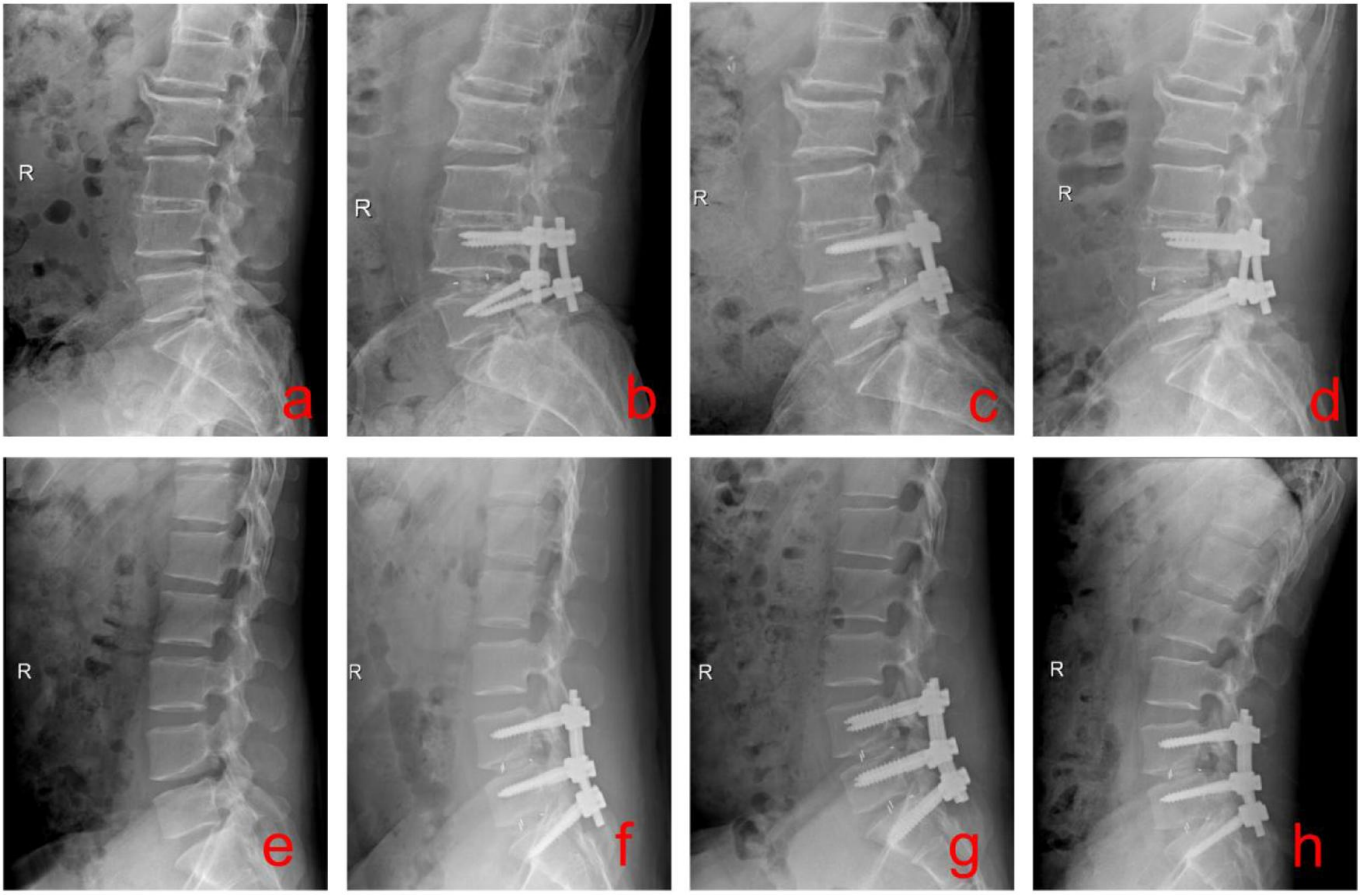
Representative cases of CM after PLIF. **(a–d)** A patient with lumbar spinal stenosis underwent L4-L5 single-level PLIF; postoperative CM occurred, and revision surgery was required after failed conservative treatment. **(e–h)** A patient with lumbar disc herniation, lumbar spinal stenosis, and L5/S1 isthmic spondylolysis underwent L4-S1 two-level PLIF; postoperative CM at L5/S1 occurred, and revision surgery was required after failed conservative treatment.

A total of 517 patients undergoing PLIF surgery were included in this study, 255 male patients and 262 female patients, with an age range of 43 to 73 years. In the postoperative follow-up, 46 patients (8.9%) developed CM, and 471 patients (91.1%) did not develop CM. Of the 46 patients with CM, all were treated symptomatically with bed rest, nutritional supplements, analgesics, and bone-building medications, 31 patients had symptomatic improvement, and 15 patients underwent revision surgery after ineffective conservative treatment. Typical patient cases are shown in Figure 1.

### Univariate analysis of CM after PLIF surgery

The univariate analysis of CM occurred after PLIF is shown in Table 1. Compared with the non-CM group, the CM group had significantly higher rates of osteoporosis, spondylolisthesis, and Modic changes, a greater PI-LL mismatch, and lower preoperative hemoglobin levels (all *P* < 0.05). No significant differences were observed in the remaining variables.

**Table 1:** Univariate analysis of CM after PLIF surgery.

Characteristic	Overall (*n* = 517)	Non-CM (*n* = 471)	CM (*n* = 46)	*P*
Age	58.49 ± 5.71	58.50 ± 5.66	58.46 ± 6.32	0.966
BMI	24.06 ± 1.44	24.03 ± 1.42	24.29 ± 1.55	0.243
Pelvic tilt	13.21 ± 2.38	13.22 ± 2.37	13.15 ± 2.48	0.845
Sacral slope	55.26 ± 8.45	55.11 ± 8.71	56.81 ± 4.94	0.193
Pelvic incidence	51.38 ± 7.06	51.06 ± 7.02	54.67 ± 6.66	0.001
Lumbar lordosis	40.97 ± 4.91	41.18 ± 4.86	38.82 ± 4.93	0.005
PI-LL	10.37 ± 6.50	9.85 ± 6.28	15.64 ± 6.39	<0.001
Time of postoperative lumbar brace use	42.19 ± 8.82	42.70 ± 8.68	36.89 ± 8.63	<0.001
Surgery time	147.55 ± 18.78	147.64 ± 18.96	146.63 ± 16.97	0.727
Intraoperative blood loss	285.17 ± 23.58	284.83 ± 23.44	288.66 ± 24.96	0.294
Preoperative WBC	5.17 ± 0.75	5.16 ± 0.75	5.29 ± 0.73	0.267
WBC day1	14.55 ± 2.47	14.56 ± 2.47	14.41 ± 2.47	0.690
WBC day3	8.06 ± 1.58	8.04 ± 1.58	8.22 ± 1.59	0.470
Preoperative Hb	134.71 ± 10.68	135.77 ± 10.09	123.84 ± 10.61	<0.001
Hb day1	118.51 ± 15.67	118.90 ± 16.14	114.47 ± 8.79	0.067
Hb day3	114.84 ± 9.56	114.78 ± 9.45	115.42 ± 10.68	0.667
Preoperative Alb	43.79 ± 1.55	43.75 ± 1.50	44.21 ± 1.93	0.058
Alb day1	35.27 ± 1.42	35.24 ± 1.39	35.61 ± 1.69	0.088
Alb day3	34.82 ± 1.74	34.86 ± 1.76	34.40 ± 1.48	0.089
Sex				
Female	262 (50.68)	241 (51.17)	21 (45.65)	0.576
Male	255 (49.32)	230 (48.83)	25 (54.35)	
Osteoporosis				
No	328 (63.44)	313 (66.45)	15 (32.61)	<0.001
Yes	189 (36.56)	158 (33.55)	31 (67.39)	
Hypertension				
No	351 (67.89)	323 (68.58)	28 (60.87)	0.366
Yes	166 (32.11)	148 (31.42)	18 (39.13)	
Diabetes				
No	369 (71.37)	342 (72.61)	27 (58.70)	0.068
Yes	148 (28.63)	129 (27.39)	19 (41.30)	
Coronary Heart Disease				
No	420 (81.24)	382 (81.10)	38 (82.61)	0.959
Yes	97 (18.76)	89 (18.90)	8 (17.39)	
Surgery Segment				
Single segment	360 (69.63)	329 (69.85)	31 (67.39)	0.858
Multisegment	157 (30.37)	142 (30.15)	15 (32.61)	
Spondylolisthesis				
No	384 (74.27)	366 (77.71)	18 (39.13)	<0.001
Yes	133 (25.73)	105 (22.29)	28 (60.87)	
Modic Change				
Non-Modic I	414 (80.08)	393 (83.44)	21 (45.65)	<0.001
Modic I	103 (19.92)	78 (16.56)	25 (54.35)	
Disc Morphology				
Non-PearShape Disc	322 (62.28)	302 (64.12)	20 (43.48)	0.009
PearShapeDisc	195 (37.72)	169 (35.88)	26 (56.52)	

WBC, white blood cell; Hb, hemoglobin; PI-LL, pelvic incidence - lumbar lordosis; Alb, albumin.

### Logistic regression of patients with CM

Using the presence of CM as the dependent variable, the statistically significant variables identified in the univariate analysis were selected as independent variables for logistic regression analysis. The results from the multivariate regression analysis indicated that: Osteoporosis(OR = 4.186, *P* = 0.003), Spondylolisthesis(OR = 8.199, *P* < 0.001), Modic change(OR = 8.279, *P* < 0.001), PI-LL(OR = 1.306, *P* < 0.001), and Preoperative Hb(OR = 0.822, *P* < 0.001) are independent predictors for CM after PLIF surgery, as presented in Table 2.

**Table 2:** Logistic regression analysis of CM after PLIF surgery.

Variables	*β*	SE	Z	*p*	OR (95% CI)
Osteoporosis					
No					Ref
Yes	1.432	0.488	2.934	0.003	4.186 (1.654–11.421)
Spondylolisthesis					
No					Ref
Yes	2.104	0.499	4.221	<0.001	8.199 (3.191–22.937)
Modic I					
No					Ref
Yes	2.114	0.513	4.123	<0.001	8.279 (3.135–23.891)
Disc Morphology					
Non-PearShape Disc					Ref
PearShapeDisc	0.774	0.484	1.601	0.109	2.169 (0.845–5.729)
Time of postoperative lumbar brace use	0.019	0.049	0.395	0.693	1.019 (0.928–1.124)
PI-LL	0.267	0.051	5.329	<0.001	1.306 (1.192–1.453)
Preoperative Hb	−0.196	0.042	−4.699	<0.001	0.822 (0.753–0.888)

### Predictive model development

**Figure 2:**
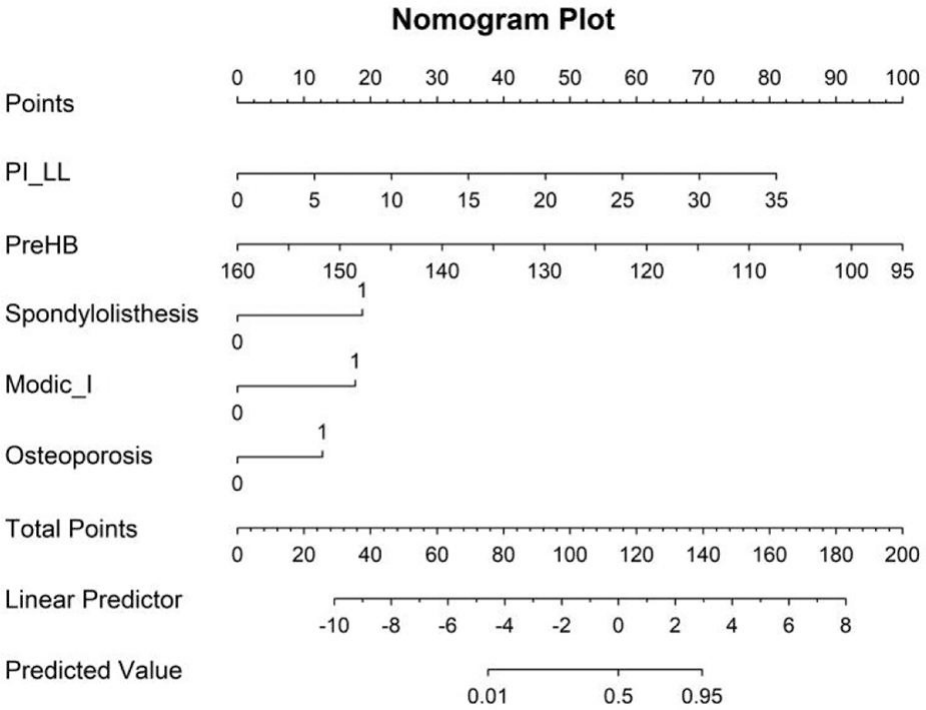
Nomogram with osteoporosis, spondylolisthesis, modic change, PI-LL, and preoperative Hb predicts the probability of CM after PLIF surgery.

Based on the results of logistic regression analysis, a predictive nomogram model for CM risk after PLIF surgery was created, incorporating five factors: Osteoporosis, Spondylolisthesis, Modic change, PI-LL, and Preoperative Hb, as shown in Figure 2.

### Discrimination and calibration of the predictive model

**Figure 3:**
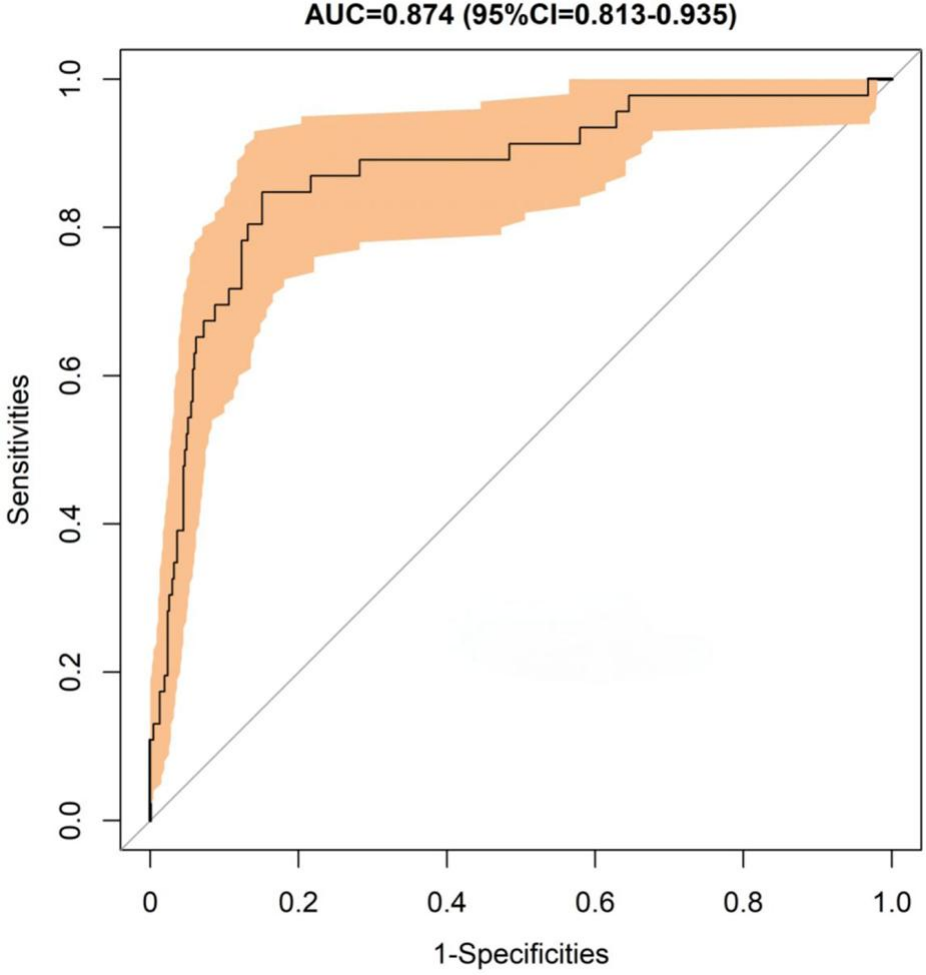
Receiver operating characteristic (ROC) curve of CM after PLIF surgery.

**Figure 4:**
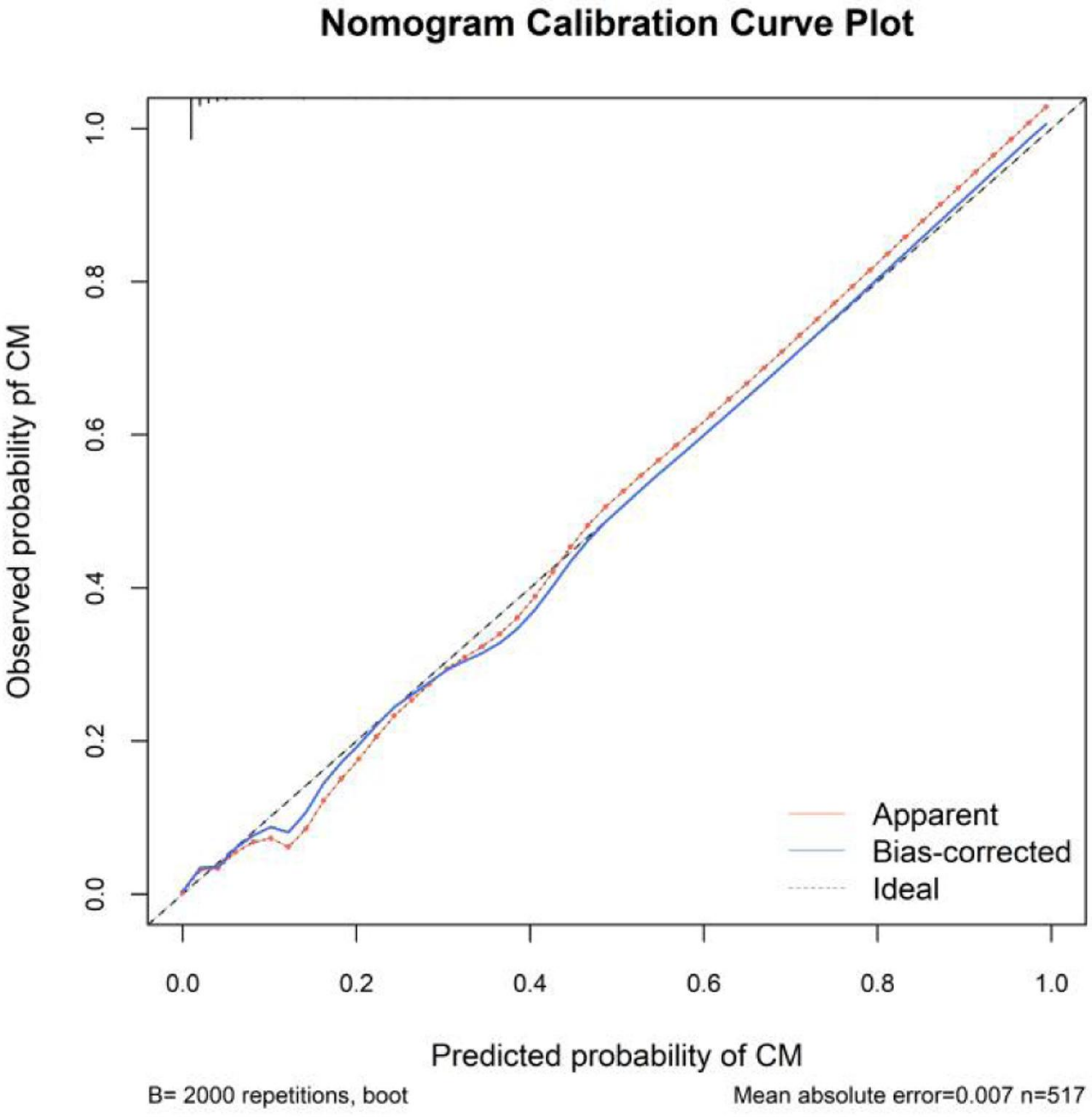
Calibration curve for predicting the probability of CM after PLIF surgery. A *P*-value of 0.481 in the Hosmer-Lemeshow test suggested agreement between the predicted probabilities and observed outcomes.

In this study, the bootstrap method was used to internally validate the nomogram model (Bootstra*p* = 2000). The area under the curve (AUC) of the receiver operating characteristic (ROC) curve of the nomogram was 0.874(95% CI: 0.813–0.935), which indicated that our prediction model showed good discrimination (Figure 3). The calibration curve of the nomogram showed good agreement between the observation value and prediction value in this dataset (Figure 4). Additionally, the Hosmer-Lemeshow goodness of fit test showed that *χ*2 = 7.530, *P* = 0.481, further indicating that the model has good calibration and acceptable predictive performance. According to ROC analysis, the optimal cut-off value of the nomogram-predicted probability was 0.112, with a sensitivity of 82.6% and a specificity of 86.2%.

### Clinical usefulness of the predictive model

**Figure 5:**
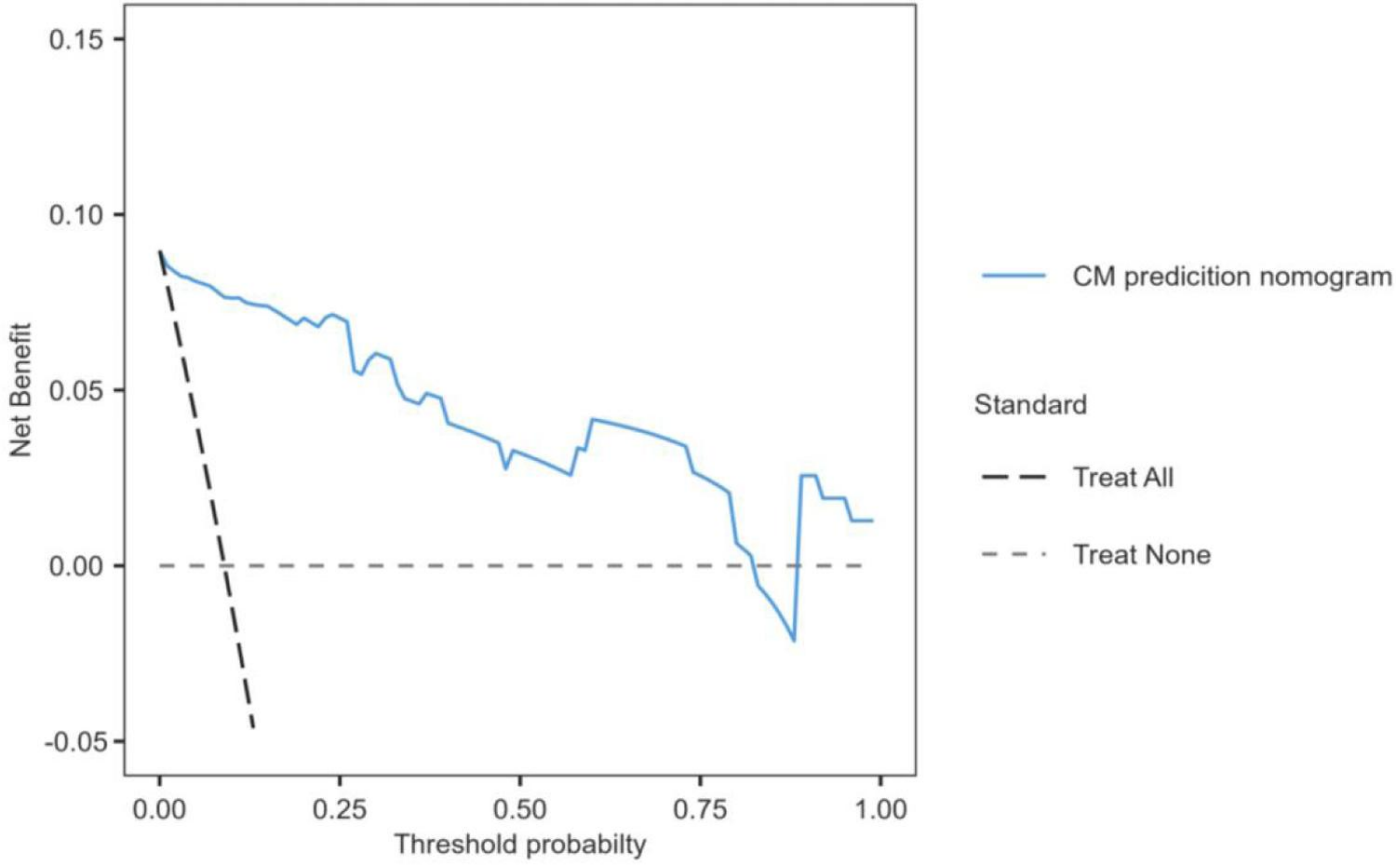
Decision curve analysis (DCA) for the CM nomogram after PLIF surgery.

To evaluate the clinical usefulness of the predictive model, a decision analysis (DCA) was performed on the data. The DCA is a novel method that assesses the clinical net benefit of the nomogram. The DCA is demonstrated in Figure 5. Decision curve analysis demonstrated that, within a threshold probability range of 0.05 to 0.81, use of the nomogram provided a greater net benefit than either the treat-all or treat-none strategies.

## Discussion

As the proportion and absolute number of elderly people in the world increase significantly, health issues have also been included in the development agenda of many countries under the aging situation. During this period, the surgical treatment of lumbar degenerative diseases has also entered the multimodal era. PLIF surgery, as a classic surgical procedure for degenerative lumbar diseases, is widely used in the surgical treatment of lumbar disc herniation, lumbar spinal stenosis, and lumbar spondylolisthesis (12). Cage implantation helps restore disc height and segmental stability and may shorten the time required to achieve interbody fusion. In recent years, with the increase in the use of PLIF surgery in the treatment of lumbar degenerative diseases, the types of complications have also increased accordingly. The neurological symptoms caused by the occurrence and protrusion of CM into the spinal canal and compression of the dura mater sac and nerve roots lead to a low quality of life after surgery. Revision surgery imposes an additional physical, psychological, and economic burden on patients. Osteoporosis (OP) is a common systemic chronic disease in middle-aged and elderly people. It is characterized by sparse trabecular structure, decreased bone density, and increased bone brittleness, which can easily lead to bone metabolism disorders (13). Previous scholars have reported that osteoporosis is one of the risk factors for the occurrence of CM through multicenter, prospective studies (14). The low bone mineralization capacity of osteoporotic patients leads to a prolonged intervertebral bone fusion time after PLIF. In addition, the difference between the elastic modulus of the pedicle screw and the bone makes the trabecular structure of the bone screw interface more prone to screw loosening under osteoporotic conditions. Before intervertebral fusion achieves a bony connection, the patient's daily activities further interfere with the formation of intervertebral trabeculae. Accordingly, patients with osteoporosis should undergo careful preoperative bone health assessment and individualized perioperative anti-osteoporosis management. For patients with poor bone quality, optimization of graft strategy and fixation planning may help reduce the risk of postoperative CM. When available, additional imaging-based assessment of bone quality may help surgeons better tailor graft strategy and fixation planning in patients with poor bone quality.

A good spinal curve can reduce the energy consumption of the muscles linked to the range of spinal motion when the human body is in a standing position. In the diagnosis and treatment of lumbar degenerative diseases, in order to enable patients to obtain better clinical prognosis, spinal sagittal sequence parameters have gradually been reported by many scholars (15, 16). The introduction of spinal-pelvic sagittal parameters helps spinal surgeons to predict the compensatory changes in the postoperative lumbar sagittal morphology when formulating surgical plans, making the formulation of surgical plans more comprehensive and rigorous, in order to achieve individualized and precise surgical treatment. Previous scholars have confirmed the correlation between pelvic posterior tilt, LL, and PI mismatch, and trunk anteversion and patients’ health-related quality of life scores through prospective studies (17). In our study, PI-LL was significantly greater in the CM group and remained an independent predictor of CM in the multivariable model (OR = 1.306, 95% CI: 1.192–1.453, *P* < 0.001). Because PI-LL was analyzed as a continuous variable, our findings suggest that each 1° increase in PI-LL mismatch was associated with a higher risk of CM. This result indicates that cage-related mechanical complications may be influenced not only by local implant-related factors, but also by the overall lumbopelvic mechanical environment. Excessive PI-LL mismatch may increase shear stress and micromotion across the fused segment, thereby adversely affecting graft-endplate interaction and early fusion stability. Therefore, in addition to adequate decompression and grafting, surgical planning should aim to restore an appropriate match between pelvic incidence and lumbar lordosis and to optimize postoperative sagittal load transfer across the instrumented segment. This interpretation is broadly consistent with previous retrospective studies. Aoki et al. reported that PI-LL mismatch was associated with greater postoperative residual symptoms after short-segment TLIF, particularly low back pain while standing, suggesting that postoperative postural imbalance has clinically relevant consequences even in short-segment fusion (18). Vazifehdan et al. further showed in a retrospective lumbar fusion series that greater PI, greater sacral slope, and a PI-LL mismatch >10° were associated with failed bony fusion, supporting the importance of preoperative and postoperative standing sagittal assessment in patients undergoing lumbar interbody fusion (19).

In recent years, hemoglobin has emerged as a critical biomarker reflecting both the oxygen-carrying capacity of the body and overall health status, with increasing recognition of its prognostic significance in spinal surgery (20). Bone regeneration and the process of interbody fusion are inherently dependent on an adequate supply of oxygen; diminished hemoglobin levels can create a hypoxic microenvironment within the intervertebral space, thereby impairing bone metabolic activity, attenuating osteoblast proliferation and differentiation, and ultimately reducing new bone formation (21). Such derangements may delay or even impede effective graft fusion. Clinical studies have shown that preoperative hemoglobin levels below the normal range (male <13.0 g/dL, female <12.0 g/dL) are associated with a significantly increased risk of suboptimal bone healing, failed graft fusion, and complications following surgery (22). Owing to the extensive exposure and substantial blood loss characteristic of PLIF procedures, patients frequently experience further postoperative declines in hemoglobin levels. Therefore, comprehensive preoperative assessment and proactive correction of low hemoglobin levels are of paramount importance for optimizing bone graft fusion and reducing the incidence of CM in patients undergoing PLIF surgery.

Lumbar spondylolisthesis is a common degenerative spinal disorder that predominantly affects elderly individuals and those engaged in long-term athletic activity (23). With the advancement of spinal surgical techniques, PLIF has become a mainstay in the management of lumbar spondylolisthesis. However, patients with lumbar spondylolisthesis exhibit a significantly higher incidence of postoperative complications following interbody fusion than those without spondylolisthesis. The overall complication rate in these patients can reach up to 30%, with CM, infection, and neural injury (24). This risk is particularly pronounced in patients with severe grades of spondylolisthesis. For instance, those classified as Meyerding grade III or IV demonstrate a markedly higher incidence of CM than patients with grade I or II disease. The reason lies in the local instability caused by spondylolisthesis, which results in increased mechanical stresses on the cage even after reinforcement with interbody fusion and internal fixation (25). These heightened forces significantly elevate the risk of postoperative CM. Therefore, lumbar spondylolisthesis should be recognized as a significant risk factor for CM after PLIF. Special attention should be paid to the prevention and monitoring of CM in both surgical planning and perioperative management for these patients to reduce the incidence of complications and optimize clinical outcomes.

Modic type I changes are primarily characterized by vertebral endplate edema and alterations in bone marrow signal, which present as hypointensity on T1-weighted and hyperintensity on T2-weighted MRI sequences. Insufficient blood supply to the vertebral endplate is considered a key factor underlying the development of Modic type I changes, as localized ischemia can lead to bone marrow edema (26). At the same time, intervertebral disc degeneration and uneven mechanical loading may cause microdamage to the endplate, further inducing inflammatory responses and bone marrow edema (27). Research indicates that Modic type I changes increase the risk of interbody CM following PLIF. This may be related to local osteoporosis and reduced fixation strength caused by bone marrow edema. Clinical evidence suggests that patients with Modic type I changes experience a higher likelihood of CM, resulting in an increased need for revision surgery (28). Therefore, the presence of Modic type I changes should be given careful consideration when planning surgical strategies and postoperative management in PLIF, to reduce the risk of CM and improve surgical outcomes and patient prognosis.

From a clinical perspective, the present findings support a more comprehensive preoperative planning strategy for PLIF. Preoperative evaluation should extend beyond the target disc level and include standing load-bearing assessment of sagittal alignment, especially spinopelvic parameters such as pelvic incidence, pelvic tilt, sacral slope, lumbar lordosis, and PI-LL mismatch. In addition, assessment of the overall sagittal profile may help identify compensatory relationships among thoracic, lumbar, and pelvic curvatures, which may influence postoperative mechanical loading and the stability of the cage-endplate construct. Segmental lordosis should also be carefully considered during cage selection, cage positioning, endplate preparation, and rod contouring, because inadequate restoration of local lordosis may contribute to unfavorable stress distribution across the fused segment. Furthermore, because osteoporosis was independently associated with CM in the present study, preoperative bone quality evaluation should be strengthened. Beyond routine osteoporosis screening, adjunctive imaging-based metrics, such as CT-derived Hounsfield unit (HU) measurement and MRI-based vertebral bone quality (VBQ) scoring, may help improve preoperative risk stratification in patients undergoing PLIF.

This study has several limitations. First, as a single-center retrospective study, it is susceptible to selection and information bias. Second, our model was only internally validated using bootstrap resampling and lacked external validation. Third, more refined imaging-based bone quality metrics, which include the CT-derived HU values and MRI-based VBQ scores mentioned earlier, were not systematically incorporated into the model and should be evaluated in future research. Despite these limitations, the nomogram showed favorable predictive performance and may serve as a convenient tool for clinical risk assessment. Further prospective, multi-center studies with external validation are warranted to enhance its generalizability.

## Conclusion

In summary, osteoporosis, PI-LL mismatch, lower preoperative hemoglobin level, lumbar spondylolisthesis, and Modic type I changes were identified as independent risk factors for postoperative CM in patients undergoing PLIF. As this was a single-center retrospective study, our findings require further validation using prediction models in future research. In addition, prospective multi-center studies with larger sample sizes are needed to verify and strengthen our conclusions

## Data Availability

The datasets generated and/or analyzed during the current study are not publicly available because they contain clinical data that may affect patient privacy, but de-identified data supporting the findings of this study are available from the corresponding author upon reasonable request.
